# The utility of the initial electrocardiogram in predicting acute coronary events in current cocaine users with chest pain in the emergency department

**DOI:** 10.3402/jchimp.v1i1.6308

**Published:** 2011-05-09

**Authors:** Celeste C.L. Quianzon, Lindsay Quade, Ishraque Shawon, Robert Ferguson

**Affiliations:** 1 Department of Medicine, Union Memorial Hospital, Baltimore, MD, USA; 2 University of Maryland School of Medicine, Baltimore, MD, USA

**Keywords:** cocaine, electrocardiogram, myocardial infarction, acute coronary syndrome, chest pain

## Abstract

**Introduction:**

Chest pain suggestive of myocardial ischemia or infarction is a common emergency department complaint and a subset of these is associated with cocaine use. It can be difficult to triage patients with chest pain while using cocaine.

**Objective:**

To assess the reliability of the initial electrocardiogram (ECG) done in the emergency department in current cocaine users suspected of acute coronary syndrome (ACS) in predicting a true event.

**Methods:**

A total of 218 charts of current cocaine users who presented with chest pain judged as possibly cardiac in nature with an initial ECG from September 2003 to August 2007 were reviewed. Initial ECG was classified into: (1) Category A (inverted T waves in two or more contiguous leads and/or characteristic ST segment elevation or depression indicative of an ischemic event possibly acute); (2) Category B (other ischemic changes but without any ST or T abnormalities such as Q wave, or bundle branch block); or (3) Category C (normal tracing, non-specific ST segment or T wave alterations).

**Results:**

Eighteen of 218 (8.3%) were confirmed to have ACS. Ten of 18 confirmed ACS patients were among the 70 cases with ECG classified as Category A. One hundred and one of 218 were Category C ECGs: five of these had ACS (three of the five had significant cardiac history) and 96 did not, consistent with 95% negative predictive value. Patients with Category A ECG characteristics were three times at risk to have ACS compared with a Category C ECG.

**Conclusion:**

The initial ECG with a good clinical history can be used effectively to triage patients presenting with chest pain and current use of cocaine in the emergency department.

Chest pain suggestive of myocardial ischemia or infarction account for more than eight million visits per year in the emergency department, a subset of which is associated with cocaine use (1). About 64,000 patients are evaluated annually in the emergency department for chest pain associated with cocaine use (2). Fifty-seven percent of these patients are admitted to rule out acute coronary syndrome (ACS) at an estimated annual cost exceeding $83 million (3, 4). Proper identification of patients at low risk for an acute coronary event is important for early discharge.

Challa and colleagues (5) evaluated the utility of the initial presenting electrocardiogram(ECG) as a tool for hospital admission determination among 258 patients who presented with chest pain in the emergency department. In this study, patients with normal ECGs are at extremely low risk for myocardial infarction while patients with ST segment changes that are not acute are at a lower risk for myocardial infection than those with acute ST segment changes. They proposed admission to the hospital based on the cardiac risk profile.

Our objective was to study similar information in the setting of cocaine use.

## Methods

We performed a retrospective study of all admissions of current cocaine users who presented with chest pain considered possibly cardiac in nature to assess the value of the initial ECG in predicting an acute coronary event. Admissions at a 325-bed community hospital in Baltimore, Maryland were identified from a database dating from September 2003 to August 2007. Inclusion criteria were the following: (1) patient presenting with chest pain possibly cardiac in nature, (2) current cocaine users defined as admission of use within the last 10 days or a urine toxicology screen positive for cocaine, and (3) presence of an initial 12-L ECG on presentation at the emergency department.

Medical records were reviewed and the following data were abstracted: demographic information, medical history, cardiac risk factors, cardiac isoenzymes, and presenting signs and symptoms. Initial 12-L ECG was obtained in all cases. Cardiac risk factors included a history of hypertension, tobacco use, diabetes, hyperlipidemia, previously diagnosed coronary artery disease, or a family history of coronary artery disease. A diagnosis of ACS was made if the patient satisfied the criteria for unstable angina, non-ST, or ST elevation myocardial infarction (6–8).

The initial ECGs were interpreted by an experienced reader who was blinded and did not have any other information on the cases. The ECG was classified into one of three categories based on a study by Brush and colleagues (9): (1) Category A – inverted T waves in two or more contiguous lead, characteristic ST segment elevation, or depression indicative of an ischemic event possibly acute; (2) Category B – ischemic changes that did not involve any ST or T abnormalities such as a Q wave or bundle branch block; and (3) Category C – normal ECG tracing or an ECG with non-specific ST segment or T wave alterations. We obtained the subsequent ECGs in the cases where the initial ECG was categorized as negative but the patient had ACS.

Data were entered in Microsoft Excel 2003 and analyzed with Epiinfo version 3.5.1. Continuous variables are resulted as mean±standard deviation. Categorical variables were reported as the percentage in frequency of occurrence. Positive and negative predictive values were calculated for Category A and C, respectively. Odds ratio with 95% confidence interval was calculated to assess the risk of ACS based on the initial ECG.

## Results

We identified 218 admissions of current cocaine users who met the criteria for the study. Patient characteristics are summarized in Table 1. The majority of the admissions were male (72%) and black (91%). The mean patient age was 49±9.2 years. Most were tobacco users (64%) and many had hypertension (68%). Fifty-eight percent (59%) had two or more cardiac risk factors. Twenty-nine percent had known coronary artery disease, 19% had diabetes mellitus, and 13% had hyperlipidemia. Five percent had family history of coronary artery disease.

**Table 1:** Patient characteristics

Patient characteristics	All (*N*=218)	ACS (*N*=18)	Non ACS (*N*=200)
Age, mean (±SD)	48.9±9.2	54.7±13.3	48.4±8.6
Gender, male *n* (%)	156 (71.6)	14 (77.8)	142 (71)
Race, black *n* (%)	199 (91.3)	17 (94.4)	182 (91)
Hypertension, *n* (%)	149 (68.3)	14 (77.8)	135 (67.5)
Smoking, *n* (%)	140 (64.2)	15 (83.3)	125 (62.5)
Diabetes mellitus, *n* (%)	42 (19.3)	1 (5.6)	41 (20.5)
Hyperlipidemia, *n* (%)	30 (13.8)	3 (16.7)	27 (13.5)
Family history CAD, *n* (%)	11 (5)	2 (11.1)	9 (4.5)
Known CAD, *n* (%)	64 (29.4)	8 (44.4)	56 (28)
Prior MI, *n* (%)	26 (11.9)	3 (16.7)	23 (11.5)
Prior positive stress test, *n* (%)	9 (4)	2 (11.1)	7 (3.5)
Prior CABG, *n* (%)	14 (6.4)	1 (5.6)	13 (6.5)
Two or more cardiac risk factors *n* (%)	126 (57.8)	13 (72.2)	113 (56.5)

Of the 218 admissions, there were 70 (32%) cases whose initial ECG was categorized as Category A, 47 (22%) cases with a Category B initial ECG, and 101 (46%) cases with a Category C ECG. Eighteen cases (8.3%) were subsequently confirmed to have ACS. Ten of the 18 patients confirmed to have ACS were among the 70 cases with a Category A initial ECG. An initial ECG with a Category A changes, thus, had a 14% positive predictive value.

**Fig. 1:**
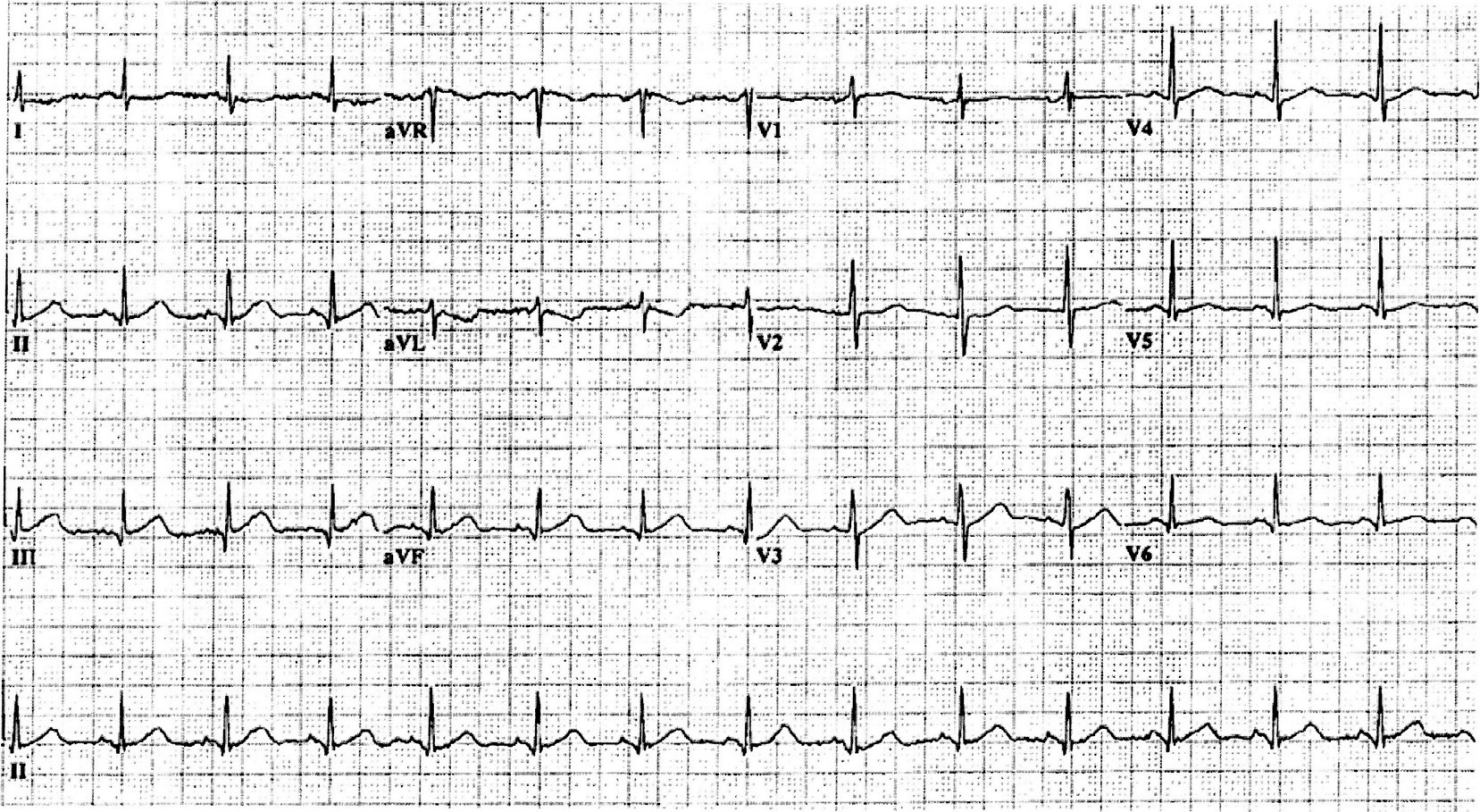
A negative initial electrocardiogram of a patient who subsequently had an ACS.

**Fig. 2:**
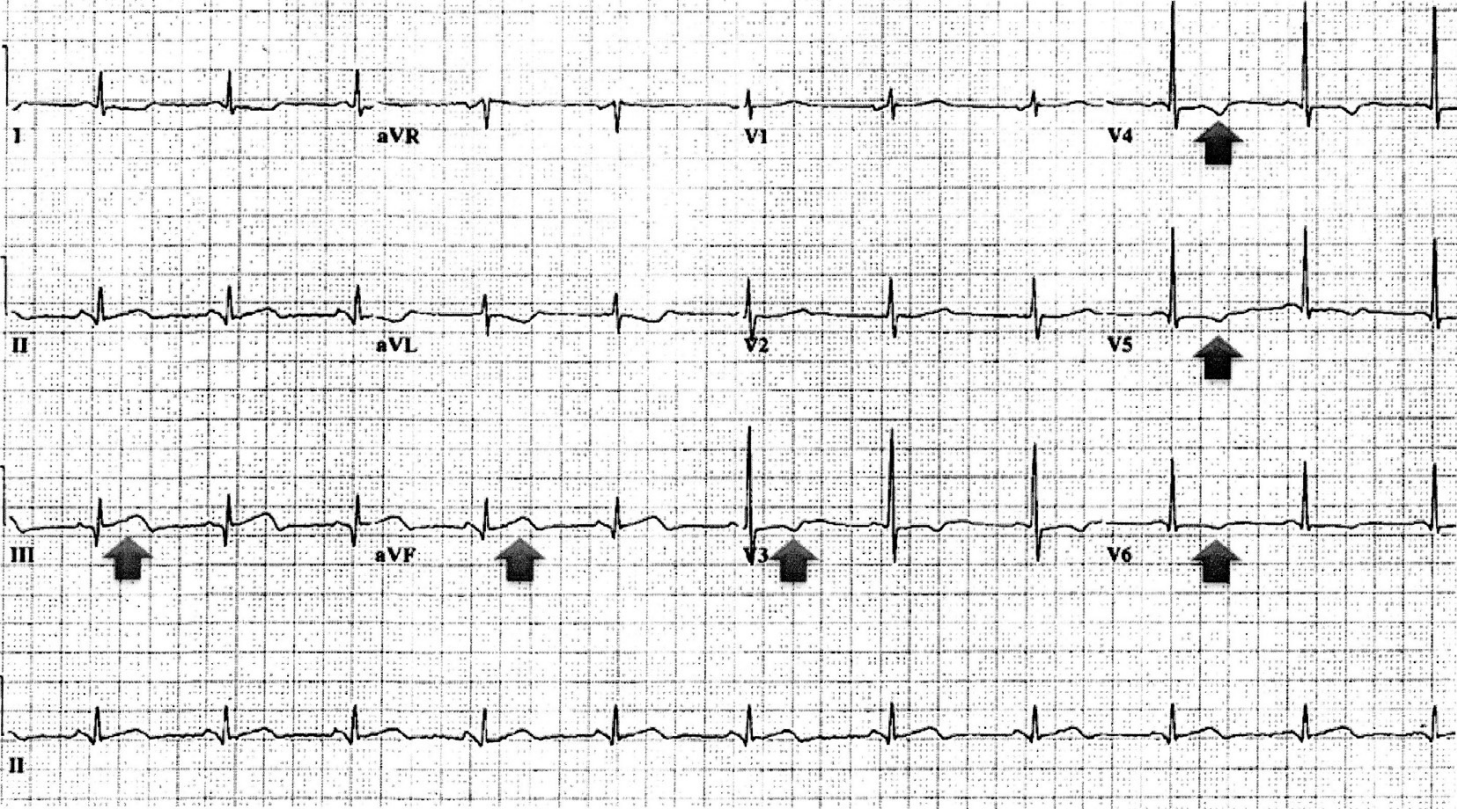
The electrocardiogram 5 hours later of the patient showing acute ischemic changes.

There were 101 of 218 cases who had a Category C initial ECG. Five patients who had a Category C initial ECG had an ACS. Interestingly, three had prior cardiac catheterization with stent placement; thus, a Category C initial ECG had a 95% negative predictive value. We were only able to obtain subsequent ECG s in four of the five cases. Four developed acute ischemic changes; two had ST segment elevation, and the rest had T wave abnormalities. One of the cases, the initial ECG shown in Fig. 1, developed acute ischemic changes 5 hours from the initial ECG (Fig. 2).

Patients with Category A ECG characteristics were 3.2 times at risk to have ACS when compared with Category C ECG (95% CI: 0.94–12.45, *p*=0.03).

## Discussion

Studies have been inconsistent regarding the incidence of cocaine-associated myocardial infarction. The incidence ranged between 0.7 and 31% (10–13). A retrospective study that evaluated 70 patients admitted for chest pain following cocaine use found a high incidence of acute myocardial infarction at 31% (12). Other retrospective studies reported a myocardial infarction incidence of 6% (11, 14) and another found an incidence of cocaine-associated myocardial infarction of 14% in 194 non-duplicate admissions (13). The Cocaine Associated Chest Pain (COCHPA) study, which is a large prospective study, showed that 6% of patients developed myocardial infarction following emergency department presentation after cocaine use (15). Our finding showed an incidence of 8.3% for cocaine associated ACS.

Identifying patients at increased risk for ACS is important to prevent morbidity and mortality. A retrospective study (13) evaluating the utility of traditional risk factors in predicting cocaine-associated acute myocardial infarction showed that patients with preexisting coronary artery disease were seven times as likely to be diagnosed than those without, and patients more than 45 years or older, hyperlipidemia, or family history of coronary artery disease are three times more at risk for cocaine-associated acute myocardial infarction than those who are not. Among the patients diagnosed with ACS in our study, 14 of the 18 cases were more than 45 years of age, 44% had known CAD, 16.7% had hyperlipidemia, and 11.1% had family history of CAD. Forty-four percent of patients with ACS had preexisting coronary artery disease compared to 28% of patients who did not have ACS. Risk factors combined with ECG interpretation appear excellent predictors in the emergency department.

Consistent diagnostic approaches are desirable to effectively triage these patients. In our study, we assessed the utility of initial ECG in predicting ACS. In prior studies of patients who present to the emergency department with chest pain, the initial ECG was found to be effective in identifying low-risk patients for myocardial infarction (5, 16). Hollander et al. (17) mentioned that the value of an ECG appeared to lack sensitivity and specificity in identifying ischemia in current users compared with older patients with ischemic chest pain from coronary artery disease. The COCHPA study (15) found a sensitivity of 36% for an initial ECG to predict a myocardial infarction. Our data showed that there were 60 of 70 cases who had Category A initial ECGs but did not have an ACS. Our data showed a sensitivity of 67% and specificity of 62% for initial ECGs with Category A findings. However, combined Category A and B findings in an initial ECG had a sensitivity and specificity of 72 and 48%, respectively.

Our data showed that an initial ECG with Category A change had a positive predictive value of only 14% for predicting an ACS, which is similar to the COCHPA study (15). Gitter et al. (18) concluded that there is a low incidence of acute myocardial infarction when the initial ECG is normal or a normal variant. Our data showed that the initial ECG has a 95% negative predictive value. If we exclude those with a significant cardiac history (three of the five), the negative predictive value would be even higher.

## Limitations

This was a retrospective chart review that was subject to limitations (i.e., no randomization, relies on medical records). We utilized a data abstraction sheet for data collection to minimize bias. The study was also limited by its relatively small sample size.

## Conclusion

The overall incidence of ACS in 218 episodes of chest pain while on cocaine in our emergency department was 8.3%. Patients presenting with cocaine-associated chest pain who have a normal initial ECG and no cardiac history had an ACS on only two occasions. Abnormal ischemic ECGs were three times more likely to result in ACS compared with normal ECGs. Our study supports the reliance of good clinical history and an initial ECG to assess chest pain in this setting.
